# First recorded case of paramyxovirus infection introduced into a healthy snake collection in Croatia

**DOI:** 10.1186/s12917-017-1015-6

**Published:** 2017-04-08

**Authors:** Jelena Prpic, Tomislav Keros, Maja Lang Balija, Dubravko Forcic, Lorena Jemersic

**Affiliations:** 1 Croatian Veterinary Institute, Savska cesta 143, 10 000 Zagreb, Croatia; 2 Centre for Research and Knowledge Transfer in Biotechnology, University of Zagreb, Rockefellerova 10, 10 000 Zagreb, Croatia; 3 Center of Excellence for Viral Immunology and Vaccines, CERVirVac, Zagreb, Croatia

**Keywords:** Snake, Paramyxovirus, Nested RT-PCR, Phylogenetic characterization, Infection, Croatia

## Abstract

**Background:**

In the present study, we describe the first paramyxovirus infection in a snake collection in Croatia caused by an introduction of new snakes that were not previously tested and didn’t show any signs of disease.

**Case presentation:**

In less than a month after introduction into a healthy colony, new snakes began to show respiratory symptoms (i.e. mouth opening, wheezing, etc.) and died within a month and a half after antibiotic therapy was applied. The same symptoms and a high mortality rate were then observed in in-contact snakes from other collections belonging to different snake families.

**Conclusions:**

Two entries of new snakes in different time periods were recorded and recognized as possible sources of infection. We stress the need for veterinary health control and monitoring of snakes prior to transportation as well as implementing obligatory quarantine measures to minimize the risk of infection among newly established snake groups.

## Background

Paramyxoviruses (PMV) are negative sensed single stranded RNA viruses with a helical nucleocapsid packaged in a pleomorphic envelope and are classified as members of the *Paramyxoviridae* family, genus *Ferlavirus* [5, 6, 11]. According to studies based on partial sequences of L, HN, and U genes [1, 11, 3], members of the genus *Ferlavirus* are grouped into three subgroups, subgroups A, B and C. There are numerous reports of *Ferlavirus* isolation worldwide from zoological and private collections. Up to now, the occurrence of PMVs has been detected in several snake (sub) families: *Colubridae, Elapidae, Viperidae, Crotalidae, Boidae* and *Pythonidae* [4, 7, 9, 11]. The purchase and introduction of new reptiles into an established collection is of high risk in regards to disease spread and manifestation as well as a probable cause of a higher mortality rate. It has been demonstrated that viral infections such as inclusion body disease (IBD) and PMV [12], as well as some bacterial infections, represent a significant risk when latently infected snakes are introduced into healthy snake collections [13].

The objective of this study was to detect PMV RNA in samples derived during 2015 from a collection of exotic snakes showing signs of disease. Furthermore, sequencing of a highly conserved fragment within PMV L-gene region was performed to define the genetic heterogeneity of the virus isolates and to compare the sequences with previously described ones from the GenBank. The aim was to retroactively define the probable sources of infection. In consensus with earlier publications [8, 10] we used a nested RT-PCR protocol targeting the L-gene [2] as the most sensitive method currently available for basic diagnostics of PMV infection.

## Case presentation

In less than a month after introduction of *Dendroaspis jamesoni jamesoni* (purchased from a private keeper from the Czech Republic in September 2014) into a healthy colony, the mamba began to show respiratory symptoms (i.e. mouth opening, wheezing, etc.) and died within a month and a half after antibiotic therapy (Marbocyl for 10 days) was applied. The same symptoms and a high mortality rate were then observed in other snakes from collections belonging to different snake families. In February 2015 the breeder purchased new snakes and introduced them into the collection. One pair (male and female) of *Dendroaspis viridis* (caught in the wild in Ghana) was purchased from a private keeper from the Czech Republic, whereas snakes *Bitis nasicornis* were purchased from Poland. *Dendroaspis viridis* didn’t show any respiratory symptoms but was in a poor condition and died within a month and a half. The snake owner noticed the same clinical signs and a high mortality rate in other snakes from his collection belonging to different snake families. Autopsy showed signs of fibrotic pneumonia.

Lung tissue (CRO-PMV-1 to CRO-PMV-6 and CRO-PMV-12) and tracheal and cloacal swab samples (CRO-PMV-7 to CRO-PMV-11) were collected (Table 1). Approximately, 0.1 g of lung samples were homogenized in 1 mL of phosphate-buffered saline (PBS; pH 7.4) and then vortexed for 1 min and centrifuged for 15 min at 1000 × *g*. Tracheal and cloacal swabs were taken by moistened cotton-tipped sterile swabs and sterile MEM-H from the snakes that began to show the signs of disease. After filtration (0.45 μm), swab samples were inoculated into a Vero cell culture and propagated for up to two passages. Vero cell culture was maintained in minimal essential medium with Hank’s salts (MEM-H), supplemented with 10% foetal calf serum (FCS) and 50 mg/ml neomycin and the incubation was carried out at 32 °C in a medium with added 2% FCS until a cytopathic effect was observed. An amount of 100 μL of each homogenized lung supernatant, swab sample and cell culture sample was used for viral RNA purification using iPrep Total RNA Kit (Invitrogen, USA) according to the manufacturer’s instructions.

**Table 1:** Animals used in the study

Host	Origin of snakes	Sample/time of death	Label
*Crotalus m. molossus*	Imported in September 2014 from Germany	Lung tissue March 2015	CRO-PMV-1
*Crotalus m. molossus*	Imported in September 2014 from Germany	Lung tissue August 2015	CRO-PMV-2
*Crotalus m. pyrrus*	Imported in 2013 from Germany	Lung tissue August 2015	CRO-PMV-5
*Dendroaspis viridis*	Imported in February 2015 from Czech Republic	Lung tissue April 2015	CRO-PMV-3
*Dendroaspis j. jamesoni*	Imported in September 2014 from Czech Republic	Lung tissue April 2015	CRO-PMV-4
*Bitis atropos*	Imported in 2012 from South Africa	Lung tissue September 2015	CRO-PMV-6
*Bitis nasicornis*	Imported in February 2015 from Poland	Lung tissue August 2015	CRO-PMV-12
*Morelia viridis*	Imported in 2009 from Croatia	Tracheal/ cloacal swab October 2015	CRO-PMV-7 CRO-PMV-8
*Crotalus l. klauberi*	Imported in 2014 from Austria	Tracheal/ cloacal swab October 2015.	CRO-PMV-9 CRO-PMV-10
*Aspidites melanocephala*	Imported in 2011 from Germany	Tracheal swab Still alive	CRO-PMV-11

The amplification of PMV L-gene fragments was adopted from previously published methodology [2]. One-step reverse transcription PCR and nested PCR assays for the amplification of 627-bp and 566-bp long products of L-gene, respectively, were performed using SuperScript III One-Step RT-PCR with PlatinumTaq (Invitrogen, USA) and PlatinumBlue PCR SuperMix (Invitrogen, USA) using primer pairs and the amplification procedure described by Ahne et al. [2]. PCR products were separated by agarose gel electrophoresis in a 1.5% agarose gel stained with ethidium bromide and visualized by UV transillumination. Twelve gel purified PCR amplicons (Wizard SV gel and PCR Clean up system, Promega, USA) derived from 10 snakes were sequenced directly (Macrogen Inc., Amsterdam, The Netherlands). To determine the phylogenetic grouping of the obtained sequences (GenBank KU207735 to KU207746) the prototype PMV sequences [11] were retrieved from GenBank. Also, similar sequences obtained using BLAST algorithm (http://www.ncbi.nlm.nih.gov) were included in the study. Sequences were aligned using ClustalX, version 2.0 [16] and analyzed by MEGA 5 [15].

## Discussion

**Fig. 1:**
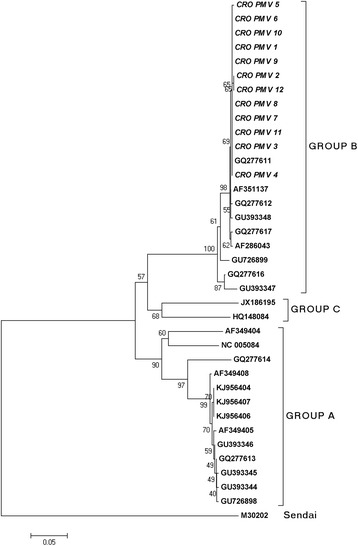
Neighbor-joining phylogenetic tree obtained by the analysis of the partial L-gene region of PMV strains derived from snake (bold, italic) samples in Croatia. Genetic distances were calculated using the Kimura two-parameter method. Genotype reference sequences (bold) were adopted from Papp et al., [11]. Bootstrap values are presented next to tree nodes. The bar represents 0.05 nucleotide substitution per site

﻿﻿The amplification of PMV L-gene region from all samples (CRO-PMV-1 to CRO-PMV-12) resulted in clear PCR products with the expected molecular size. The phylogenetic relationship analysis of the obtained sequences showed that all analyzed PMV sequences grouped into group B (Fig. 1) of the proposed genus *Ferlavirus* [11]. Sequences CRO-PMV-1, CRO-PMV-3, CRO-PMV-4 and CRO-PMV-6 to CRO-PMV-11 were found to be 100% identical among themselves in the 443 nt L-gene region, while sequences CRO-PMV-2, CRO-PMV-12 and CRO-PMV-5 differed from these sequences in one nucleotide on positions 241 and 413, respectively. Nucleotide differences in 443 nt long region coding for 143 aa long polypeptide caused differences in aa composition (aa position 81 and 138). The valine replacement by the phenylalanine residue at position 81 of the 143 aa long L-polypeptide doesn’t show any effect on protein structure and function, since both of these amino acids are hydrophobic. However, the replacement of hydrophilic glutamine by the basic arginine residue at position 138 could have an impact on viral characteristics, as has been observed in Newcastle disease virus when the replacement of glutamine by the arginine residue in F protein reduced viral replication and attenuated the virus pathogenicity [14].

All Croatian PMV isolates showed high nucleotide identity with sequences of PMV isolates from Germany (GQ277611, AF351137, GQ277612, GU393348 and GQ277617) and Austria (AF286043).

According to the data gained, there are two possible sources of infection. The first source of infection was *Dendroaspis jamesoni jamesoni* (CRO-PMV-4) imported from the Czech Republic in September 2014. The disease spread to *Crotalus molosus molosus* (CRO-PMV 1). Virus sequences isolated from these snakes are mutually identical. The second sources of infection were *Dendroaspis viridis* (CRO-PMV-3) and *Bitis nasicornis* (CRO-PMV-12) that were introduced into the collection in February 2015. We can presume that the disease spread from *Bitis nasicornis* (CRO-PMV-12) to *Crotalus molosus molosus* (CRO-PMV-2) since the virus sequences isolated from these snakes are mutually identical. However, the source of infection for the strain isolated from *Crotalus molosus Pyrrus* (CRO-PMV-5) could not be identified. We presume that point mutations could be initiated by multiple passages in vivo originating from previously introduced clusters since this snake was in the colony since 2013. The passages in Vero cells couldn’t be responsible for the observed mutations, since the sequences from swab samples and cell culture supernatants were 100% identical.

## Conclusions

Our results imply that the infection was introduced into the healthy collection by new snakes that were not previously tested or showing signs of disease. Since no vaccine is presently commercially available, basic veterinary health checks and quarantine measures are of great importance to minimize the risk of infection among newly established snake groups.
